# Supplementary material for: No Effect of Continuous Transcutaneous Auricular Vagus Nerve Stimulation on the P3, the P600, or Physiological Markers of Noradrenergic Activity in an Oddball and Sentence Comprehension Task
Source: Psychophysiology. 2026 Feb 12;63(2):e70258. doi: 10.1111/psyp.70258 (PMC12896093; doi:10.1111/psyp.70258)

Supplementary material

Figure A1.

*Trial-wise ERP amplitudes and participant-wise condition means for the (**A**) P3, (**B**) syntactic P600, and (**C**) semantic P600. Each point represents a single trial (jittered for visibility), and larger filled circles indicate the mean amplitude for each participant in each stimulation condition. Lines connect the means of the two stimulation conditions within participants. Panels are faceted by stimulus type (Standard vs. Oddball). Plots are provided for descriptive purposes and to illustrate the relative magnitude of inter-trial and inter-individual variability in the data entering the mixed-effects models.*

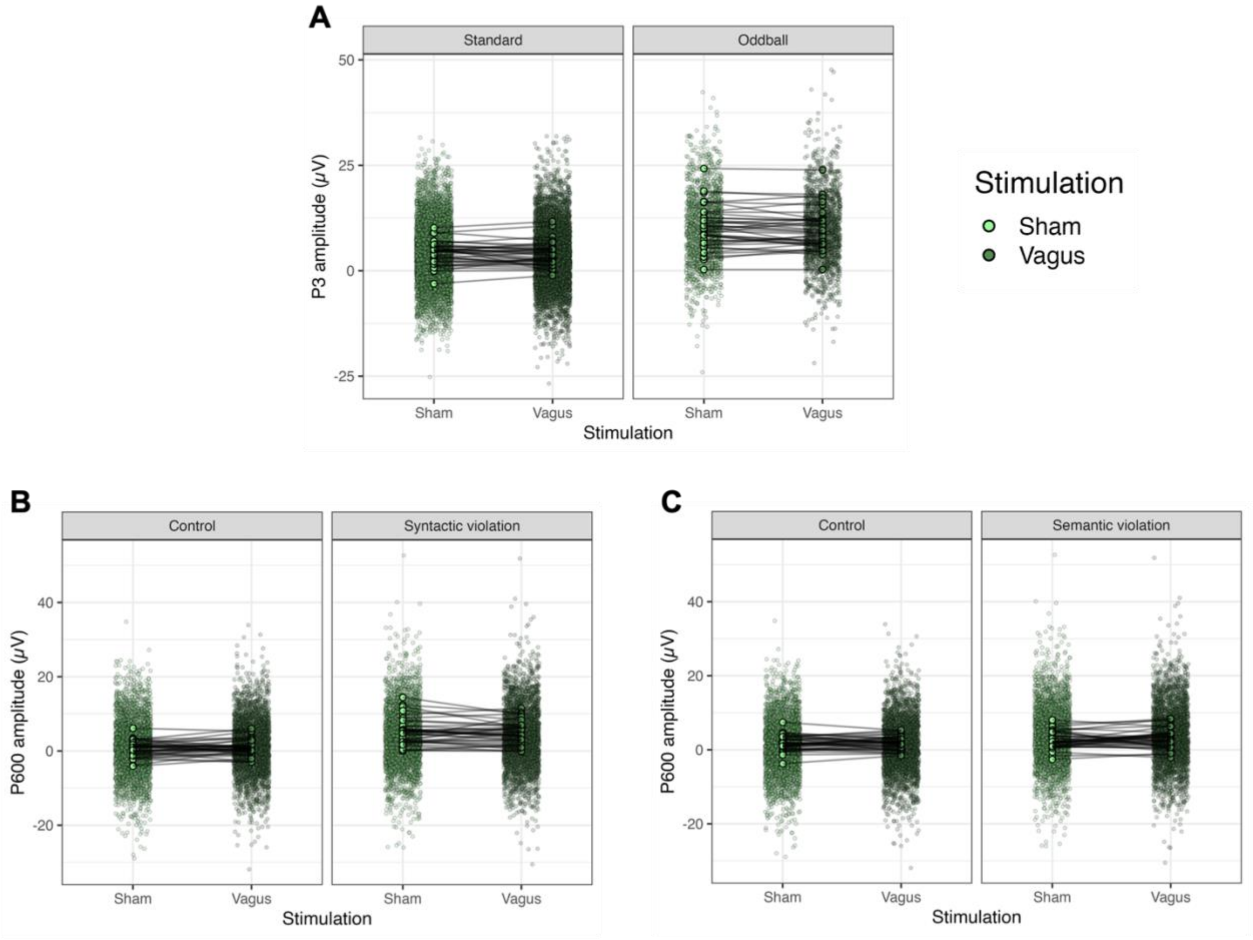

Supplement: Supplementary file 1 — Data S1: psyp70258‐sup‐0001‐supinfo.pdf. [file PSYP-63-e70258-s001.pdf]
